# Fairly rare clear cell adenocarcinoma mimicking liver cancer: a case report

**DOI:** 10.1186/s40792-018-0500-x

**Published:** 2018-08-16

**Authors:** Norifumi Harimoto, Kei Hagiwara, Takahiro Yamanaka, Norihiro Ishii, Takamichi Igarashi, Akira Watanabe, Norio Kubo, Kenichirou Araki, Hayato Ikota, Masafumi Suyama, Takeshi Maki, Shinichi Aishima, Hiroyuki Kuwano, Ken Shirabe

**Affiliations:** 1 Department of General Surgical Science, Division of Hepatobiliary and Pancreatic Surgery, Graduate School of Medicine, Gunma University, Maebashi, Japan; 2 Department of Human Pathology, Graduate School of Medicine, Gunma University, Maebashi, Japan; 3 Maki Hospital, Takasaki, Japan; 4 Department of Pathology and Microbiology, Faculty of Medicine, Saga University, Saga, Japan; 5 Department of General Surgical Science, Graduate School of Medicine, Gunma University, Maebashi, Japan

**Keywords:** Liver, Ovary, Clear cell carcinoma, Hepatectomy

## Abstract

**Background:**

Clear cell carcinoma commonly occurs in the ovary and kidney, and clear cell cholangiocarcinoma was rarely reported. Differential diagnosis which the origin of the tumor located on the liver surface is intrahepatic or extrahepatic was difficult. Herein, we report a case of clear cell adenocarcinoma mimicking liver cancer.

**Case presentation:**

This was a 55-year-old female who had the tumor with cystic component in the liver. She was performed hepatectomy and diagnosed as clear cell adenocarcinoma. Histopathological evaluation revealed intra-cystic clear cell adenocarcinoma. The tumor has ductal structure including mucin and atypical nuclear with clear cytoplasm. The tumor was separated from the liver and the diaphragm. The expression of Pax8 was positive, but the expression CK7 and HNF1β was positive and that of CD10 and ER was negative, which indicate that the tumor has the feature of clear cell carcinoma of ovary, not renal cell carcinoma nor cholangiocarcinoma.

**Conclusions:**

Our experience with this patient suggests that this tumor may originate from the endometriosis onto the diaphragm from the detailed results of immunohistochemical staining.

## Background

Clear cell carcinoma is generally thought to originate from ovary and kidney [1]. Clear cell carcinoma of the ovary is composed of glycogen-containing clear cells and hobnail cells [1, 2]. Clear cell carcinoma of the ovary also shares many similarities with renal clear cell carcinoma [3]. Primary renal cell carcinoma is sometimes implanted to the ovary or peritoneum [4]. Additionally, clear cell cholangiocarcinoma [5] and peritoneal clear cell carcinoma [6, 7] were also rarely reported. Although recent genomics research will reveal the difference of these carcinomas, differential diagnosis of the primary site is difficult.

Herein, we report a case of clear cell adenocarcinoma mimicking liver cancer.

## Case presentation

**Fig. 1:**
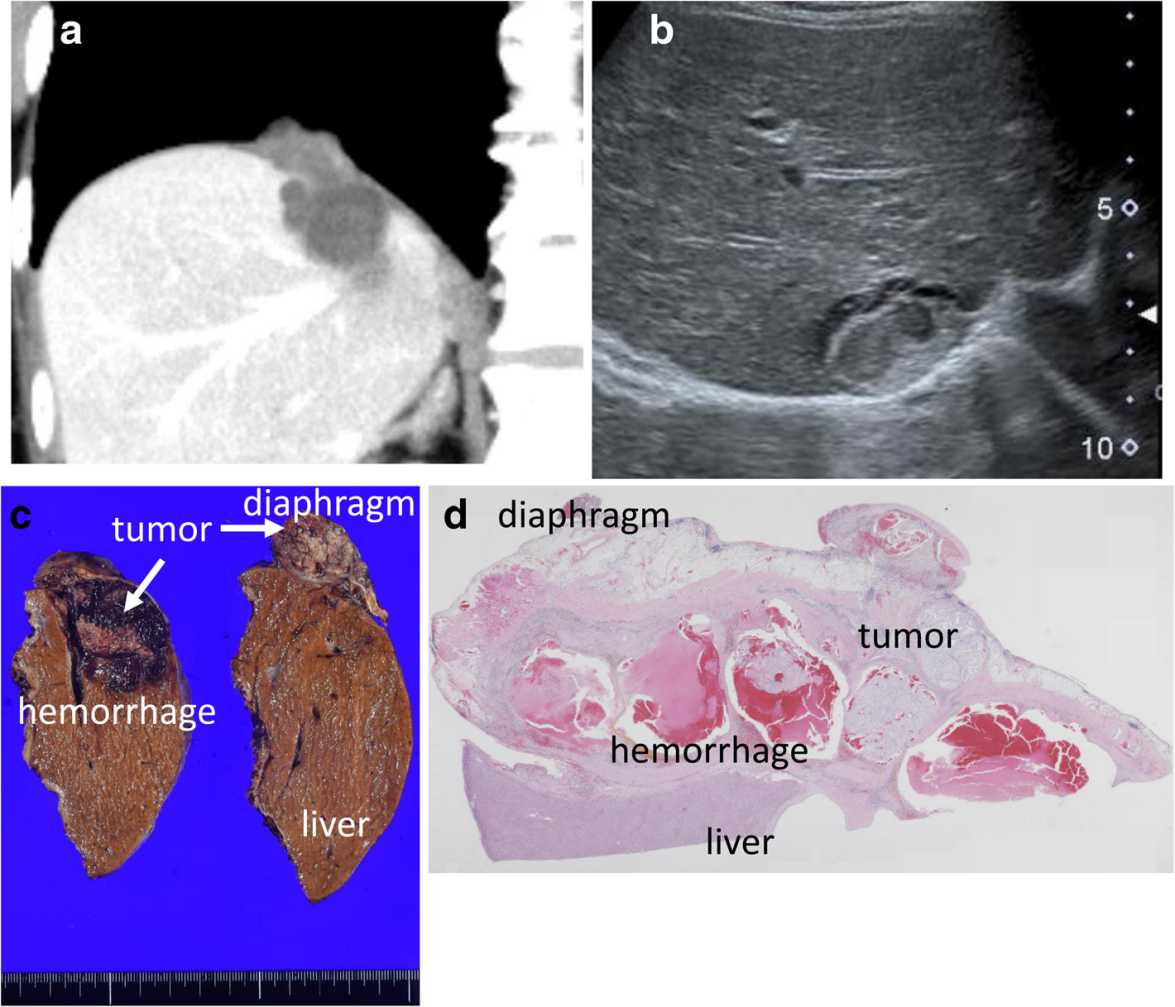
Imaging. **a** US shows the tumor was located within the cyst. **b** CT shows the cystic lesion at segment 8 of the liver. The tumor was protruding to the diaphragm. **c** Macroscopic findings revealed the tumor and hemorrhage within the cyst. **d** Microscopic findings revealed the tumor and hemorrhage within the cyst. CT, computed tomography; US, ultrasound sonography

A 55-year-old woman regularly visited our hospital as an outpatient because of hepatitis B occult infection. A liver tumor was point out by CT. CT revealed a protruding liver tumor located at segment 8 3 cm in size, which include cystic lesion (Fig. 1a). US and MRI reveal the same feature (Fig. 1b). There was no distant metastasis. The patient had no past or family history including gynecological illness. 18F-FDG PET revealed the accumulation of 18F-FDG, and maximum standard uptake value was 2.3. Laboratory results included a white blood cell count of 3200/μL and platelet count of 189,000/μL. Prothrombin time international normalized ratio was 1.02. Total serum bilirubin was 0.9 mg/dL, direct bilirubin 0.03 mg/dL, albumin 4.5 g/dL, aspartate aminotransferase 22 U/L, alanine aminotransferase 17 U/L, alkaline phosphatase 187 U/L, and gamma-glutamyltranspeptidase 49 U/L. Tumor markers such as CEA, CA19-9, AFP, and DCP were normal. HBs-antigen and HBc-antibody were positive, and HBs-antibody and HCV-antibody were negative. The Child–Pugh score was 5, grade A. She was diagnosed as intrahepatic cystadenocarcinoma and received extended posterior segmentectomy including diaphragm. Macroscopic findings revealed the tumor buried to the liver with the intracystic hemorrhage (Fig. 1c). The protruded comportment was closely touched to the diaphragm. Microscopic findings revealed the tumor and hemorrhage within the cyst (Fig. 1d). Tumor was located between the liver and diaphragm.

**Fig. 2:**
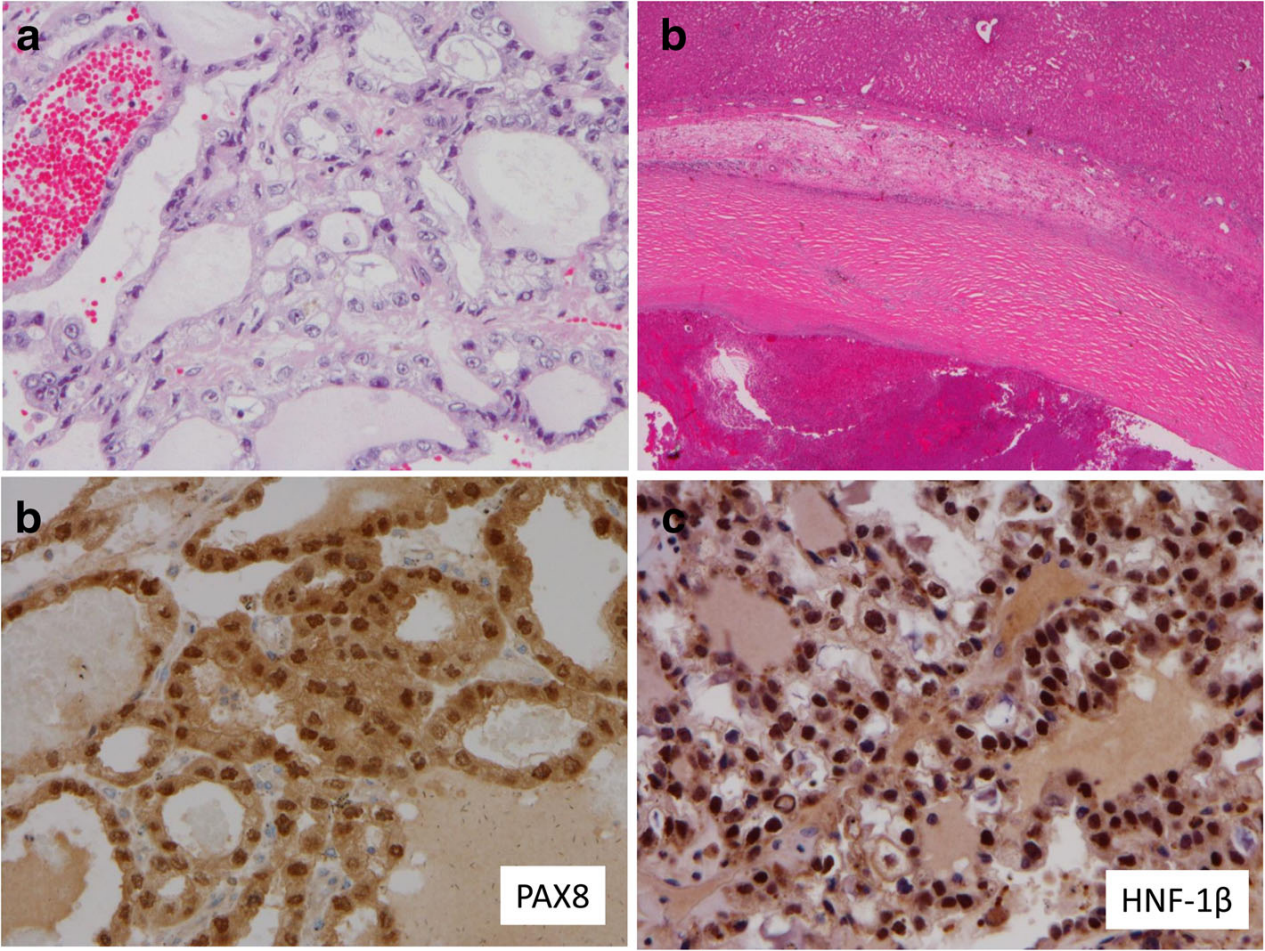
Histopathological findings. **a** The tumor has ductal structure including mucin and atypical nuclear with clear cytoplasm (H&E stain). **b** The tumor was separated from the liver and the diaphragm. **c** The expression of Pax8 was positive. **d** The expression of HNF1β was positive. H&E, hematoxylin and eosin; Pax8, paired box gene 8; HNF1β, hepatocyte nuclear factors 1β

Histopathological evaluation revealed intracystic clear cell adenocarcinoma. The tumor has ductal structure including mucin and atypical nuclear with clear cytoplasm (Fig. 2a). The tumor was separated from the liver and the diaphragm (Fig. 2b). There is no traffic with the bile duct and ovarian stroma. PAS staining was positive. There was lack of ovarian clear cell carcinoma’s features such as hobnail appearance. The expression of Pax8 (Fig. 2c) was positive, but the expression CK7 and HNF1β(Fig. 2d) was positive and that of CD10 and ER was negative, which indicate that the tumor has the feature of clear cell carcinoma of the ovary, not renal cell carcinoma nor cholangiocarcinoma. The patient was discharged 13 days after surgery with no complication. According to the microscopic findings, gynecological interview and examinations after hepatic resection were performed, but there were no sign of menstrual irregularity and genital bleeding, and no endometriosis.

## Conclusions

Clear cell carcinoma is characterized by clear cells containing glycogen arranged in tubular, papillary, and solid patterns, which commonly occurs in the ovary and kidney [1, 2]. On the other hand, clear cell cholangiocarcinoma was rarely reported [5]. Differential diagnosis which the origin of the tumor is intrahepatic or extrahepatic was difficult in this case. Microscopic findings revealed membrane of cyst was separated from the liver and the diaphragm. In this case, there was no malignant tumor in the ovary or kidney and there was no evidence of endometriosis at the peritoneum. Fujiki et al. reported a case of diaphragmatic clear cell carcinoma in a patient with a medical history of ovarian endometriosis [8]. The clear cells and hobnail cells were observed in the diaphragmatic tumor. The hobnail cells were not in this case, but the immunohistochemical stain such as PAX8 and HNF1β. Pax8 is a crucial transcription factor for organogenesis of the thyroid gland, kidney, and müllerian system and is highly found in highly ovarian and renal clear cell carcinomas [9]. HNF1β is also a transcription factor involved in glucose homeostasis and anti-apoptosis, and recent immunohistochemical studies have shown that it is frequently and highly expressed by ovarian clear cell carcinoma [10]. The great majority of ovarian clear cell carcinomas reported as a HNF-1β-positive and ER-negative immunoprofile [11]. CK7 was the subtype of cytokeratin, and CD10 was a cell surface enzyme with neutral metalloendopeptidase activity. These were used for the differential diagnosis of renal clear cell carcinoma [12]. In renal clear cell carcinoma, CK7 is negative and CD10 is positive. In ovarian clear cell carcinoma, CK is positive and CD10 is negative, which is similar to this case.

Primary peritoneal clear cell carcinoma (PPCC) is very rare. It was firstly described in 1990 [6], and so far, only 11 cases have been reported in the English literature [7]. The differential diagnosis between PPCC and ovarian clear cell carcinoma was difficult.

We previously reported that rare metastases which appear to be buried in the liver parenchyma on US or CT were closely touched under the diaphragm [13]. In this case, the tumor was not invaded to both liver and diaphragm mimicking liver cancer. That may be implanted from ovarian endometriosis onto diaphragm.

In conclusion, we presented a case of clear cell carcinoma mimicking liver cancer. We suspected that this tumor may originate from the endometriosis onto the diaphragm from the detailed results of immunohistochemical staining.

## Abbreviations

18F-FDG-PET: 2-Deoxy-2-[fluorine-18]fluoro-d-glucose integrated with computed tomography
AFP: α-fetoprotein
CK: Cytokeratin
CT: Computed tomography
DCP: Des-γ-carboxy prothrombin
ER: Estrogen receptor
MRI: Magnetic resonance imaging
PAX8: Paired-box gene 8
US: Ultrasound sonography
